# Stratigraphic reassessment of Grotta Romanelli sheds light on Middle-Late Pleistocene palaeoenvironments and human settling in the Mediterranean

**DOI:** 10.1038/s41598-022-16906-9

**Published:** 2022-08-08

**Authors:** Pierluigi Pieruccini, Luca Forti, Beniamino Mecozzi, Alessio Iannucci, Tsai-Luen Yu, Chuan-Chou Shen, Fabio Bona, Giuseppe Lembo, Brunella Muttillo, Raffaele Sardella, Ilaria Mazzini

**Affiliations:** 1 Dipartimento Di Scienze Della Terra, Università Di Torino, Via Valperga Caluso, 35, 10125 Turin, Italy; 2 Dipartimento Di Scienze Della Terra “A. Desio”, Università Degli Studi Di Milano, Via L. Mangiagalli 34. 20133, Milan, Italy; 3 Consiglio Nazionale Delle Ricerche (CNR), Istituto Di Geoscienze E Georisorse, Via G. Moruzzi 1, 56124 Pisa, Italy; 4 Dipartimento Di Scienze Della Terra, “Sapienza” Università Di Roma, Piazzale Aldo Moro 5, 00185 Rome, Italy; 5 Marine Industry and Engineering Research Center, National Academy of Marine Research, Kaohsiung, 806614 Taiwan, ROC; 6 High-Precision Mass Spectrometry and Environment Change Laboratory (HISPEC), Department of Geosciences, National Taiwan University, Taipei, 10617 Taiwan, ROC; 7 Research Center for Future Earth, National Taiwan University, Taipei, 10617 Taiwan, ROC; 8 Museo Civico Dei Fossili Di Besano, Via Prestini 5, 21050 Besano, Varese, Italy; 9 Ministero Dell’Istruzione, Ferrara, Italy; 10 Dipartimento Di Studi Umanistici, Università Di Ferrara, Corso Ercole I d’Este, 32, 44121 Ferrara, Italy; 11 Consiglio Nazionale Delle Ricerche (CNR), Istituto Di Geologia Ambientale E Geoingegneria, Area della Ricerca di Roma 1, 00015 Monterotondo, Rome, Italy

**Keywords:** Geology, Geomorphology, Palaeontology, Sedimentology, Archaeology

## Abstract

During the last century, Grotta Romanelli (Southern Italy) has been a reference site for the European Late Pleistocene stratigraphy, due to its geomorphological setting and archaeological and palaeontological content. The beginning of the sedimentation inside the cave was attributed to the Last Interglacial (MISs 5e) and the oldest unearthed evidence of human occupation, including remains of hearths, was therefore referred to the Middle Palaeolithic. Recent surveys and excavations produced new U/Th dates, palaeoenvironmental interpretation and a litho-, morpho- and chrono-stratigraphical reassessment, placing the oldest human frequentation of the cave between MIS 9 and MIS 7, therefore embracing Glacial and Interglacial cycles. These new data provide evidence that the sea reached the cave during the Middle Pleistocene and human occupation occurred long before MISs 5e and persisted beyond the Pleistocene- Holocene boundary.

## Introduction

**Figure 1:**
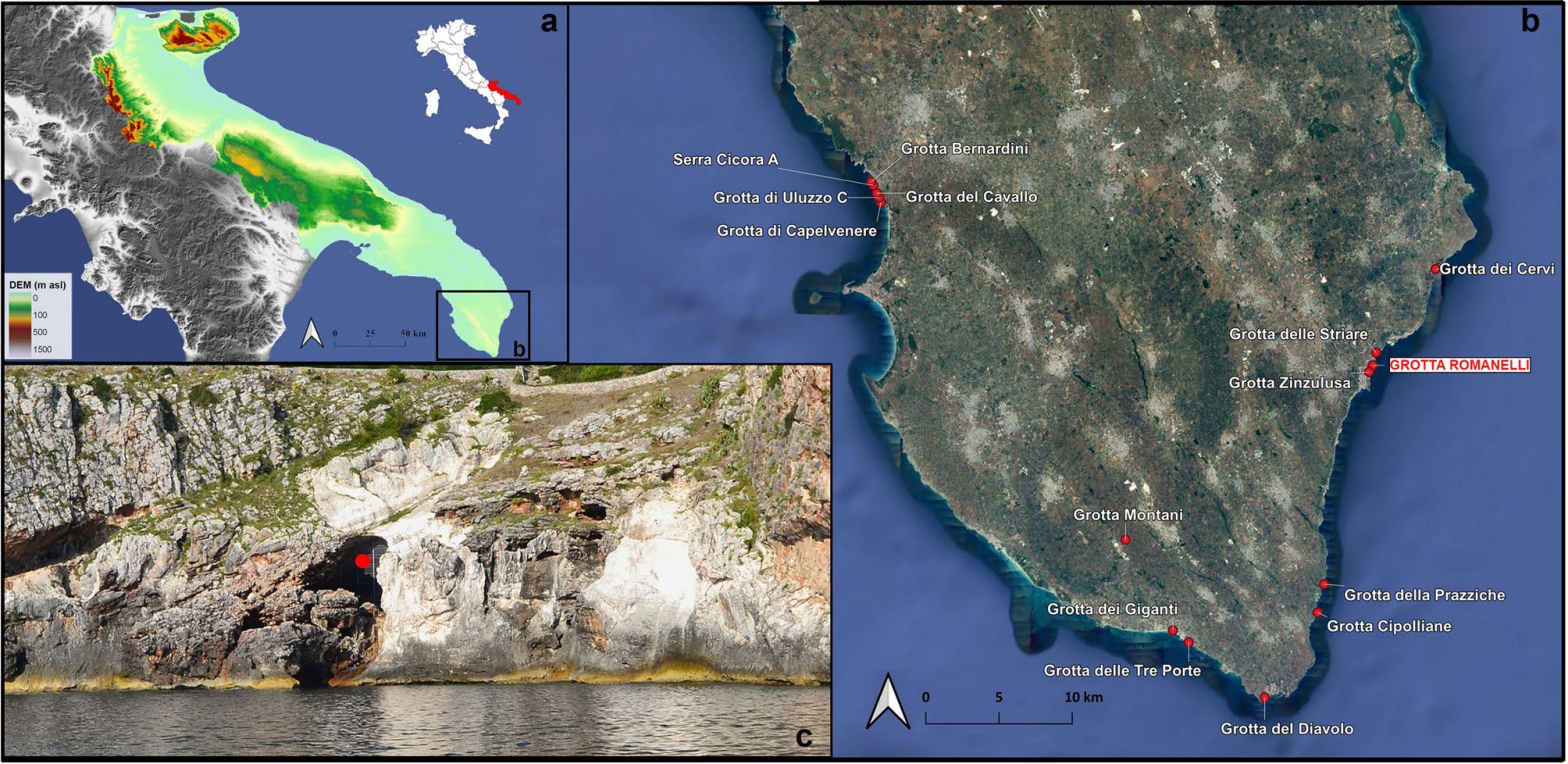
Geographic position of Apulia (map of Italy modified from https://it.wikipedia.org/wiki/File:Italy_map_with_regions.svg); Digital Elevation Model of Apulia in southern Italy (elaborated with QGIS 3.16.7 software and free downloaded from https://tinitaly.pi.ingv.it/)^17^ (**a**) Satellite image view of the Salentine Peninsula (south-eastern Apulia) (visualised and elaborated within QGIS 3.16.7 software with “QuickMapServices” plugin https://nextgis.com/blog/quickmapservices/) with the known caves filled by Middle to Late Pleistocene deposits (caves position free downloaded from http://www.catasto.fspuglia.it/) (**b**) The entrance to Grotta Romanelli opening into the limestone cliff (red dot) (**c**) Maps elaboration and photo by L. Forti.

The Italian territory is rich in stratigraphic records from caves and rock shelters bearing archaeological findings that have contributed to the framework of the Quaternary stratigraphy in Europe. Recent research on iconic Italian sites revealed the importance of their chrono-, litho-, morpho- and bio-stratigraphical reassessment, considering the progresses that stratigraphy, palaeontology, and archaeology have made in the last 100 years^1–5^. In the Apulian region (Southern Italy) (Fig. 1a), several caves and rock shelters formed in a typical Mediterranean karst landscape^6^ preserved important records of long-term environmental change and prehistoric human activity^6–8^. Many of these caves are facing the seashore nowadays and their litho- and morpho-stratigraphical records provide information about Quaternary sea-level history^9–13^, palaeoenvironments^14^ and their relationships with human settlements. These caves also record direct evidence of Homo neanderthalensis King, 1864 (i.e., Grotta di Lamalunga^15^) and the earliest European occurrence of Homo sapiens Linnaeus, 1758 (i.e., Grotta del Cavallo^16^) (Fig. 1b).

Grotta Romanelli opens into Cretaceous limestone along the cliffs of the southeastern coast of Apulia (Fig. 1c). Since the beginning of the twentieth century, Blanc and others systematically excavated and described the infilling deposits of the cave and its content (reference therein in^18,19^) (Supplementary Material 1, Fig. S1 in Supplementary Material). The cave soon became a reference for Quaternary geomorphological and geological studies for the assessment of the Last Interglacial Relative Sea Level (RSL) in this area of the Mediterranean^9,20^. The deposits infilling the cave and the RSL indicators, such as Marine Limiting Points (MLPs,^21,22^) made of tidal notches, *Lithophaga* burrows, algal encrustation and shoreface deposits, were referred to MISs 5e marine highstand^21,22^. Within the cave, the MLPs are well preserved as they are buried by a succession made of, from bottom to top, sandy pebbles beach deposits, angular breccia and the sequence of the so-called “Terre Rosse” and “Terre Brune”, silty-sands deposits originally considered of aeolian origin^23,24^. The chrono-stratigraphical framework proposed by the first researchers was supported by two U/Th dates on stalagmitic layers and nine radiocarbon dates on charcoal and humic acids^25–27^. Following the correlation to MISs 5e for the beginning of the sedimentation inside the cave, the impressive fossil record of birds and mammals, together with portable art and parietal engravings, hearts, and limestone or flinty lithic tools was referred to the time span MIS5–1, also documenting the Last Glacial Maximum with the finding of the iconic Great Auk *Pinguinus impennis* (Linnaeus, 1758) (= *Alca impennis*)^23,28^. Moreover, Grotta Romanelli has been for a long time considered a reference site for early Late Pleistocene European and southern Italy terrestrial ecosystems, mammal faunal correlations and Middle Palaeolithic human technology (e.g.,^29–33^ and reference therein).

The archaeological and palaeontological findings from Grotta Romanelli, hosted in museums and institutions across Italy, have been the subject of several studies, both confirming^29,30^ or questioning^31,34,35^ the chrono-stratigraphic setting proposed by Blanc^23^. Recently, new excavations led to a reassessment of the stratigraphy of the uppermost part of the cave infilling^36^ and a partial revision of some of the palaeontological remains^33,37–39^. A critical review of the Last Interglacial MLPs and Sea Level Points Indicators (SLIPs in 20) along the stable coasts of the Mediterranean Sea^11^, indicated the highest Grotta Romanelli’s tidal notch (tn) as older than MIS 5e.

Here we present new U/Th ages, coupled with new litho- and morpho-stratigraphical evidence from the 6 m thick sequence still preserved inside the cave and the sedimentary record and geomorphological features found along the cliff in its immediate surroundings, revising the age of the lowermost cave infilling and the palaeoenvironmental interpretation proposed by Blanc^23,24^. Moreover, for the first time, we present evidence of litho- and morpho-stratigraphical units on the cliff above e below the cave entrance, which, bounded to the chrono-stratigraphical data of the cave deposits, provide new information for the Middle Pleistocene RSL history of this area of the Mediterranean.

## Results

**Figure 2:**
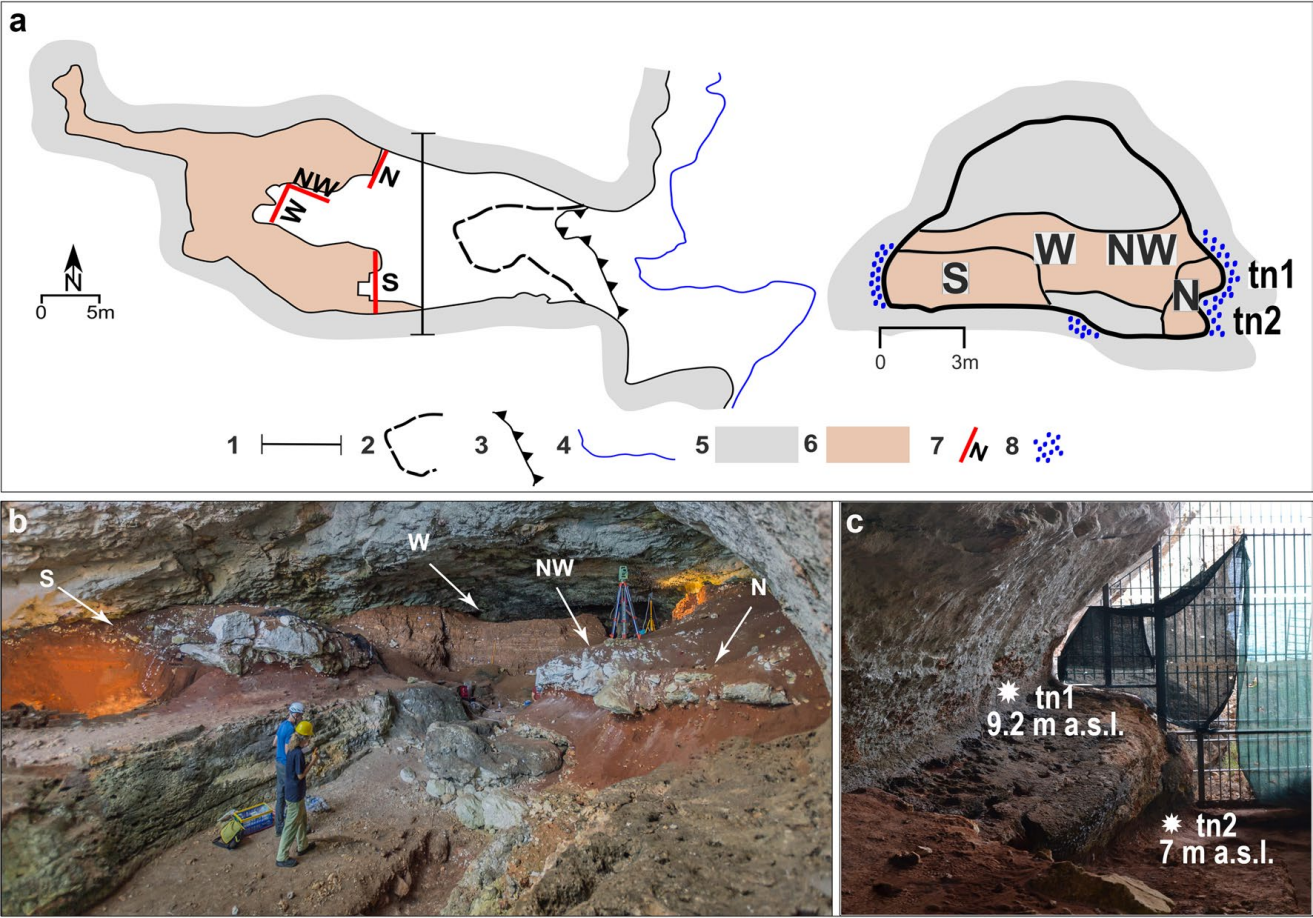
Planimetry of the cave and cave cross section. Legend: 1 Cross section; 2 Cave ceiling limit; 3 Cliff; 4 Sea level; 5 Bedrock; 6 Sedimentary cave filling; 7 Sections location and orientation; 8 *Lithophaga* burrows (**a**). Overall view of the cave from the northern side with the location of the described sections (**b**). The two tidal notches (tn1 and tn2) carved into the bedrock and observable on the northern side of the cave, originally buried by the sedimentary succession (**c**). Planimetry modified from 17 by P. Pieruccini and L. Forti. Artwork and photos by L. Forti and P. Pieruccini.

**Figure 3:**
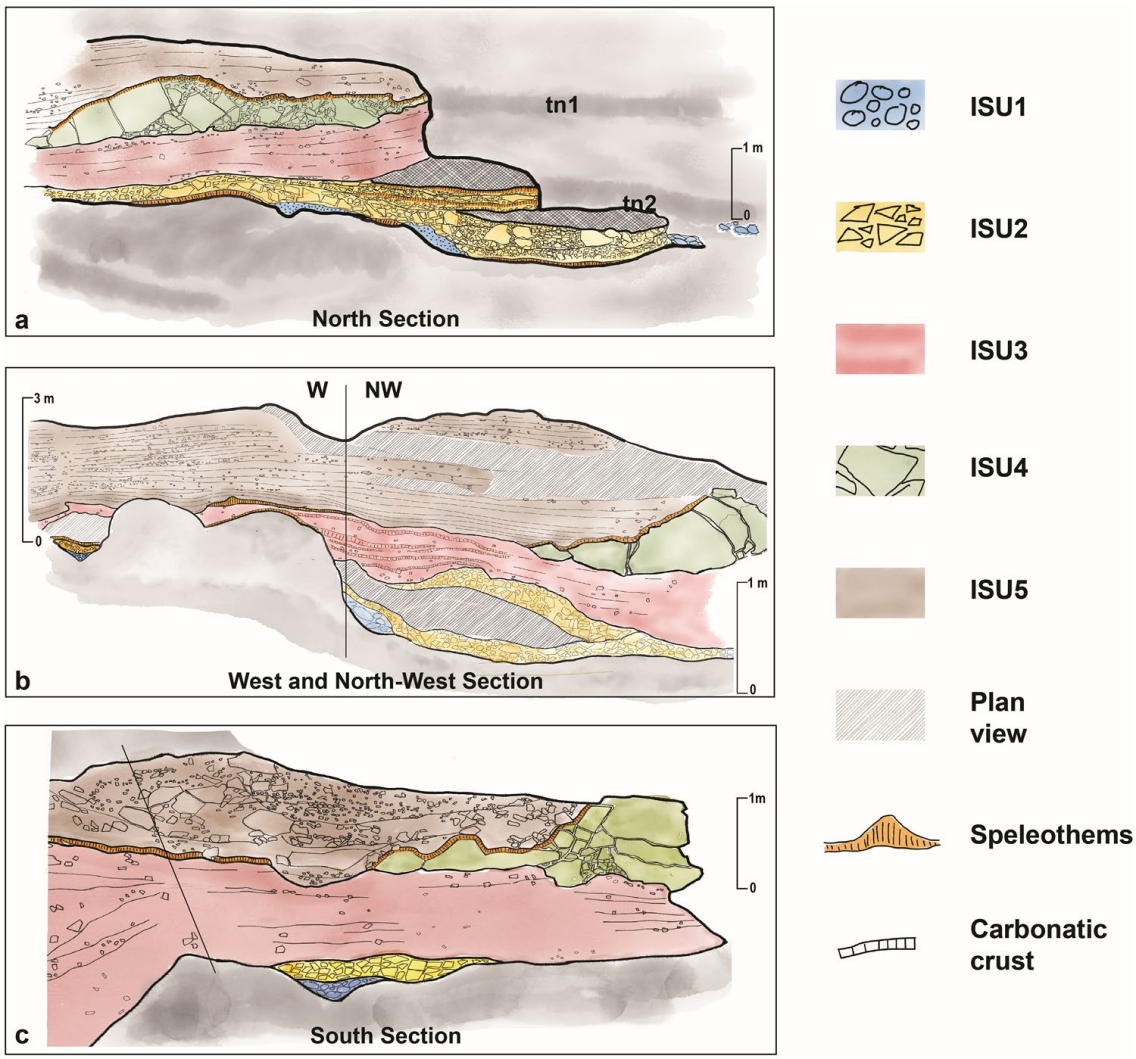
Line drawings of the Sections (for reference to orthophotos see Fig. S2 in Supplementary Material)*.* Acronyms refer to Table 1. Artwork by P. Pieruccini.

During the 2017–2021 campaigns, four sections were opened inside the cave for stratigraphical and sedimentological observations coupled with geochronological (U/Th) and micromorphological sampling (Table 1, Supplementary Material 2, Fig. S2, S3 and Table S1 in Supplementary Material). On those sections, North (N), South (S), North-West (NW) and West (W) (Table 1, Fig. 2), five stratigraphic units were recognised (Table 1, Fig. 3). New geomorphological survey outside the cave was carried out. Six stratigraphic units were identified outside the cave along the cliff at higher or lower elevation or partially covering the cave entrance.

**Table 1:** Sedimentary characteristics of the stratigraphic units (SU). Prefix I for SUs’ inside the cave, prefix O for SUs’ outside the cave. The abbreviations of archaeological and palaeontological findings refer to the new excavation, except when in bold (literature record). Legend: A—Aves; Am—Amphibia; Bt—Bone tools; C—Crustacea; E—Echinodermata; Ft—Flint tools; For—Foraminifera; H—Hearts; Hr—Human remains; Lt—Limestone tools; M—Mammal; mMal—marine Malacofauna; cMal—continental Malacofauna; Ost—Ostracods; P—Pisces; Pa—Portable Art; R—Reptilia. (References^23,24,34,35,38–42^).

Stratigraphic units this work	Main sedimentary characteristics	Stratigraphic units Blanc (1920)	Archaeological findings	Fossil findings
**INSIDE THE CAVE**				
**ISU5**	Thinly to medium layered sands, silts and clays with stone lines and lenses of matrix-supported angular to subangular fine to coarse-grained monogenic limestone gravels	**A,B,C,D,E**	**Bt, Ft, H, Pa**	**A,** Am, E, **Hr, M,** cMal, mMal, **P,** R
**ISU4**	Roofspall made of open-work angular to subangular monogenic limestone boulders, cobbles and gravels, whose voids are infilled by sediments coming from the overlying ISU5. The top of ISU4 is locally draped by a flowstone up to 8 cm thick	**F**		
**ISU3**	Clays and silts with variable amounts of fine to coarse-grained quartz sands and rare scattered fine to coarse angular to subangular monogenic limestone strongly weathered gravels. In Section S, W and NW the top of this unit is locally draped by a discontinuous flowstone (Level F of Blanc, 1920) from a few mm to several cm thick. In section NW irregular impregnative carbonate crusts are also present	**G**	**Lt**	**A,** Am, E, **M,** cMal, **P,** R
**ISU2**	Wavy bedded unsorted angular to subangular open-work to matrix supported coarse-to very-coarse grained limestone gravels, pebbles and boulders, with dark brown to yellowish brown sandy-silty matrix. Very rare rounded to sub rounded pebbles. The top of this unit is locally characterised by the presence of an irregular flowstone (Level H of Blanc,1920) whereas other flowstones and impregnative carbonate crusts locally also cap the lower beds	**H**	**H, Ft, Lt**	**A, Am,** C, E, M, cMal, **mMal,** P
		**I**		
**ISU1**	Rounded to subrounded polygenic limestone gravels, pebbles and boulders with variable amounts of sandy-silty matrix, resting over the bedrock. Locally, it contains rounded to subrounded re-worked pumices. Occurrence of foraminifera, ostracoda and molluscs	**K**	H, **Lt**	For, **mMal,** Ost
**OUTSIDE THE CAVE**				
**OSU6**	Thinly bedded breccia dipping seaward up to 30° made of fine- to coarse-grained, angular to subangular, strongly cemented limestone flake-shaped gravels, open-work to matrix supported			
**OSU5**	Roughly bedded breccia dipping seaward up to 40° made of unsorted, coarse- to very coarse-grained, strongly cemented, mostly open-work limestone boulders, pebbles and gravels, unconformably overlying OSU4 and OSU3			
**OSU4**	Roughly horizontally bedded or slightly seaward dipping, unsorted angular to subangular, strongly cemented limestone gravels, cobbles and boulders, from open work to matrix supported. Rounded to sub rounded pebbles and gravels are found in the lowermost beds that bury unconformably the underlying OSU3. Some of the boulders are made of OSU2			M
**OSU3**	Roughly bedded and strongly cemented conglomerates made of unsorted, subrounded to rounded and subangular polygenic limestone gravels, cobbles and boulders resting over an almost flat abrasion platform carved on the bedrock between ca. 3,8 m a.s.l. and 4,8 m a.s.l			
**OSU2**	Gently dipping bedded and cemented breccia made of unsorted coarse to very coarse, monogenic, angular to subangular limestone gravels, pebbles and boulders, from open work to matrix supported			M
**OSU1**	Packed to cemented bedded conglomerates made of massive, unsorted, polygenic, coarse-to very coarse rounded to subrounded, unsorted polygenic limestone gravels, pebbles and cobbles. They bury a flat or slightly undulated basal surface carved on the bedrock and to the south is unconformably overlaid by OSU2

The litho- and morpho-stratigraphical settings of the successions inside and outside the cave are herein described, providing information on sedimentary environments and their palaeoenvironmental significance. Stratigraphic units are named with the prefix I or O indicate Inside or Outside the cave, respectively (Table 1).

### Cave litho-, morpho-stratigraphy and related sedimentary environments

**Figure 4:**
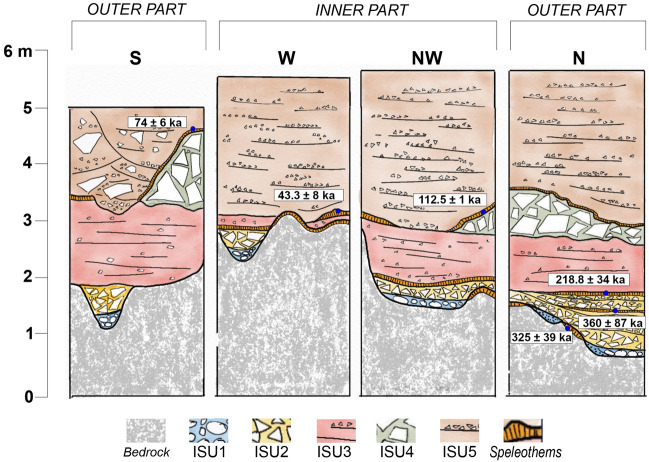
Summary of the succession filling Grotta Romanelli and U/Th dates. The location of the sections refers to in Fig. 2b. Artwork P. Pieruccini.

Today, the infilling sedimentary succession is cropping out only in the innermost part of the cave due to the impressive volume of sediments excavated by Blanc and afterwards (Fig. 2a, Supplementary Material 1). Originally completely filling the cave, the succession shows differences in thickness, geometry, and sedimentary facies according to the sectors of the cave where the sections are opened and the morphology of the bedrock (Fig. 2b). The basal part of the sediments fills solution and erosional features formed earlier on the floor and on the sides, both in vadose and subaerial conditions when the cave was already open to marine ingression. These features, undercutting the bedrock, consist of: (1) a basal erosional scour that enters the cave for about 25 m from the entrance, large max 2 m and getting narrower toward the inner part and (2) two tidal notches (tn), more evident on the northern side of the cave. The higher tidal notch (tn1) is found at 9.2 m a.s.l. and the lower (tn2) at 7 m a.s.l. Both tidal notches are characterised by the presence of algal encrustation and abundant *Lithophaga* burrows (Fig. 2c). The lithostratigraphic setting can be broadly subdivided into two parts: coarse- to very coarse-grained limestone gravels, pebbles, and boulders in the lowermost part of the succession abruptly changing upwards to fine-grained silts, clays and sands containing lenses of angular to subangular fine- to coarse-grained limestone gravels. A wedge-shaped roof spall is interbedded within the finer grained part of the succession (Fig. 3, Fig. 4). The main bounding surfaces are mostly horizontal or undulated. U/Th datings were performed on speleothems (flowstones) at the bottom and embedded within ISU2 (Fig. 4). The results provided ages of 325 ± 39 ka on top of the bedrock and 360 ± 87 ka and 218.8 ± 34 ka (Table S1 in Supplementary Material) in the lower-mid part and the top of the Unit respectively. In the N and S sector of the cave, ISU3 is buried under a roof spall (ISU4 in Fig. 3) covered by a flowstone U/Th dated at 112.5 ± 1 ka (N sector, Fig. 4, Table S1 in Supplementary Material) and 74 ± 6 ka (S sector, Fig. 4, Table S1 in Supplementary Material). Finally, the stalagmite on top of ISU3 in the W sector, provided a U/Th age of 43.3 ± 8 ka (Fig. 4, Table S1 in Supplementary Material).

### Out of Cave Morpho- and Litho-stratigraphy

**Figure 5:**
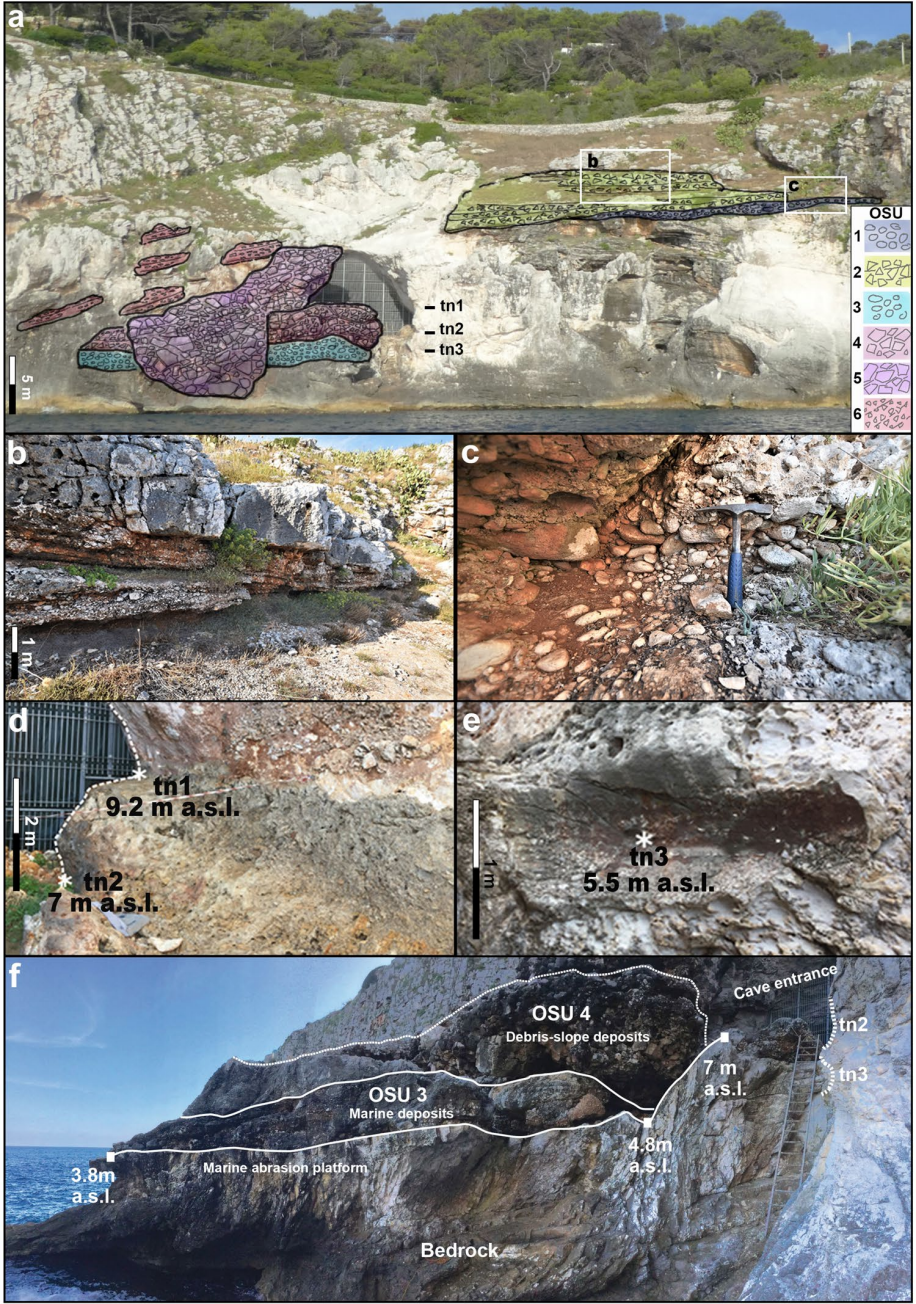
Frontal view of the cave entrance, surrounding cliff and geometry of the O-SU units (**a**). Detail of OSU2 (**b**). Detail of OSU1 (**c**). Detail of tidal notches tn1and tn2 (**d**); detail of tidal notch tn3 (**e**). View of the different clastic deposits partially covering the marine abrasion platform and the entrance of the cave (**f**). OSU units refer to Table 1. Artwork and photos by P. Pieruccini and L. Forti.

Outside the cave, both in the upper and the lower part of the cliff, further evidence of morpho- and lithostratigraphic units have been observed and described (Table 1, Fig. 5). They are shoreface deposits related to RSL highstands, older and younger than those observed within the cave. In both cases, the shoreface deposits are buried under terrestrial limiting points (TLPs sensu 20) debris-slope deposits indicating RSL lowstands. On the cliff next to the cave entrance, three tidal notches are preserved, tn1 at 9.2 m, tn2 at 7 m and tn3 at 5.5 m a.s.l. (Fig. 5a). In detail, tn1 and tn2 are the out-of-cave continuations of the tidal notches observed within the cave, where they have a lateral continuity of about 15 m (Fig. 5d). At the cave entrance, tn1 and tn2 are continuous, the higher is wider and deeper (1.4 and 0.7 m) than the lower (1 m  and 0.4 m) and their morphology suggests their formation during rising RSL^43–45^. They are also characterised by a continuous riddling by *Lithophaga* holes, whereas only tn1 preserves a vermetid reef. Tn3 is found only below the cave entrance, laterally preserved for about 1.5 m high, 1.1 m wide and 30 cm deep, and it is partially covered by small patches of cemented reddish matrix gravels whereas to the south it is almost completely buried by OSU3. Above Grotta Romanelli, in the northern sector of the bay, a conglomerate made of unsorted polygenic pebbles and cobbles (OSU1) bury a flat erosional surface on the bedrock at about 18 m a.s.l. (Fig. 5a). and is unconformably overlain by OSU2 made of an ossiferous cemented angular breccia (Fig. 5a).

## Discussion

New fieldwork at Grotta Romanelli allowed a litho-, morpho- and chronostratigraphical re-assessment of the successions inside and outside the cave. This achievement is of paramount importance since the former chronostratigraphical setting of Grotta Romanelli assumed the beginning of the sedimentation inside the cave during MIS 5e and became a paradigm for Quaternary archaeology, palaeoanthropology, palaeontology, stratigraphy and sea-level history of Italy and the Mediterranean^23^. In Grotta Romanelli, the sedimentary evidence points to the deposition within an active humid karst setting^7^. Moreover, the position of the cave relative to the sea increases its importance concerning the sea-level change history of this sector of the Mediterranean^22,46–48^. In fact, the position of the water table in the coastal zone is linked to eustatic sea-level changes^49^ and the succession within Grotta Romanelli is one of the older in-cave marine sedimentary records not eroded by high rates of water flow. After the works of Blanc ^23,24^ (Supplementary Material 1) the RSL indicators within the cave (tn1 and tn2 in Figs. 2, 3) were considered coeval with and associated to the shoreface deposits belonging to ISU1 and were attributed to the Last Interglacial. This morpho-stratigraphical assumption was based on the observation that these were the only RSL indicators higher than the present-day sea level along the cliff of Grotta Romanelli. ISU1 is the only sedimentary MLP marker within Grotta Romanelli where sandy and clayey sediments were preserved in small conservative features, such as potholes, whereas gravels, pebbles and cobbles entered the cave during high-energy events resting over tn2, the lower tidal notch. Outside of the cave, above and below the cave entrance, three MLPs are for the first time found and described: OSU1 located at ca. 18 m a.s.l. (Fig. 5a, c), OSU3 found at ca. 3.8 m a.s.l. (Fig. 5a, f)  and tn3 at 5.5 m a.s.l. (Fig. 5e, f). Therefore, MPLs found within Grotta Romanelli cannot be uniquely associated with the Last Interglacial. U/Th ages of ISU2 provide the older geochronological constraint for the whole succession. In fact, this chronology indicates that ISU1 should be dated back to the Middle Pleistocene. ISU2 is made of autochthonous debris derived from rock degradation of the ceiling and walls or from short-distance mass-deposition, re-worked within the cave according to the floor morphology lacking any palaeoenvironmental markers such as freeze–thaw features or frost slabs. Partial transport by water run-off is also suggested by the rough trough cross bedding and by the rare presence of reworked rounded marine pebbles from the underlying ISU1. Periods of non-deposition are indicated by the wavy irregular flowstone on top of the beds (Fig. 3) and by the local presence at the bottom of phytoclastic beds, possibly related to local small ponds with standing carbonate-rich water. The presence and distribution of sediments belonging to ISU2, therefore, does not provide specific palaeoclimatic nor palaeoenvironmental indications being mainly associated with the morphology of the cave^7,50^. The deposition of ISU2, according to the U/Th dates, occurred across a long-time span, suggesting that during this part of the Middle Pleistocene the cave was almost empty and that it underwent scarce, mainly autochthonous depositional events, also incorporating the older evidence of human and animals’ frequentation of the cave, including fire features, limestone lithic tools and burnt and unburnt bones^23,24^. During the last decade, a few fossils of the historical collection recovered by Blanc have been revised and the rhino material from ISU1 and ISU2 was referred to S. *hundsheimensis*^35^. Depositional environment and dynamics suddenly changed after ISU2, which is paraconformably buried by ISU3, macroscopically characterised by reddish colour (“Terre Rosse”). ISU3 is made of thinly layered planarly or cross-bedded silts, clays and sands indicating aqueous deposition through low-energy runoff. The lack of any evidence of in situ clay illuviation or other long-term soil-formation related features suggests that the iron-enrichment and the carbonate leaching of the groundmass is derived from the erosion of leached, argillic limestone soils in the overlying plateau and surrounding slopes. In fact, the sandy and silty mineral fraction of ISU3 is made primarily of quartz suggesting its provenance from the erosion of aeolian deposits such as coastal dunes or other older deposits originally present on top of the plateaux (i.e. OSU1) or from colluvial sediments or soils covering the surrounding landscape. The sediments therefore have been washed into the cave system and transported by run-off and/or karstic waters, indicating the beginning of landscape destabilisation^51^ within a warm and humid climatic context as suggested also by the presence of pollen spectra with a consistent amount of olive tree^52^. Once arrived inside the cave the sediments were redistributed over short distances by bi-directional water flows both toward the internal and the external part of the cave, by means of predominant sheet-flows (plane parallel bedding) and occasionally within rills (local cut and fill), possibly related to events of increased water availability (i.e. strong rainfall) alternating with periods of standing water (fining-upward trends and laminated clays) (Fig. S3d in Supplementary Material). Contemporaneous physical weathering of the bedrock within the cave provided the coarse-grained autochthonous carbonate fragments. Although the lack of evidence of dense vegetation, phases of non-deposition are highlighted by the common presence of biological voids, including chambers and channels (Fig. S3c in Supplementary Material), burrowing features, faecal pellets and locally small-sized coprolites. Rare elements of anthropogenic origin (i.e., burnt bone fragments) and fire-related origin (charcoals and charred plant tissues) (Fig. S3a, Fig. S3b in Supplementary Material) also suggest a short-distance transport of anthropogenic deposits within the cave system. Assuming that the age of the speleothem might reflect a long period of non-deposition within the cave and dipping vadose waters with precipitation of calcite, these ages post-date the deposition of ISU3 at least at the beginning of the Late Pleistocene. Due to the “colluvial” nature of the sediments inherited by the erosion of Interglacial leached soils ISU3 can be correlated to the early phases of climate deterioration that followed the Last Interglacial (i.e., MISs 5d-b). Fossils from ISU3 were recently debated and rhino remainss were attributed to *Stephanorhinus hemitoechus*^42^, canid specimens ascribed to *Canis lupus*^34^ and otter material to *Lutra lutra*^39^. ISU3 and ISU4 are in turn buried by ISU5, the so-called “Terre Brune '' (due to their brownish colours) of Blanc^23^ (Fig. 3, Fig. 4). The sedimentary characteristics are very similar to those of ISU3 and point to deposition in a low-energy sheet-wash and runoff environment alternating with phases of standing water within local ponds formed by consecutive events (Fig. S3e, Fig. S3f in Supplementary Material), although the lack of major discontinuities within the succession suggests that it was probably laid down at a regular pace. The random distribution of cross bedding dip suggests a direction of sediments transport according to local irregular morphology, i.e., the top of ISU4 that dammed the inner part of the cave, where finer grained sedimentation prevailed, and concentrated the coarser grained deposition in the southernmost sector of the cave (Sect. S in Fig. 3, Fig. 4) where a broad and shallow channel was filled by coarser-grained sediments including bedrock boulders and cobbles. Similarly, to ISU3, there is no evidence of soil formation features and the composition suggests the re-working of both ISU4 (limestone gravelly fraction) and ISU3 (colluviated reddish clays, Fig. S3g in Supplementary Material). The overall brownish colour and the lack of iron sesquioxide’s precipitation, together with the abundance of plant tissues, also suggests a sediments’ source from the erosion of brown soils covering the slopes surrounding the cave washed within the cave. Moreover, the abundance of anthropogenic components, such as charcoal fragments, bones (Fig. S3h in Supplementary Material) locally with evidence of thermal impact, charcoals and charred plant remains, indicate the reworking of anthropic deposits from other sectors of the cave^33^. However, most of the anthropogenic components are reduced in size and with seldom evidence of weathering or secondary impregnation by phosphate (Fig. S3g in Supplementary Material) and their origin should be related to short-distance re-working within the cave, lacking any features that could be linked to trampling or crushing. Moreover, radiocarbon dates of the same Unit in the S, W and SW sectors of the cave, provided ages for its deposition between 13.6 cal ka BP and 11.4 cal ka BP^36,53^ indicating high sedimentation rates at the Pleistocene-Holocene transition. Such rapid sedimentation occurred by both erosion of the surrounding landscape and related sediments and soil covers and partial re-working of the older succession within the cave, including the autochthonous coarse-grained gravels and cobbles of bedrock produced by the degradation of the cave walls and ceiling.

**Figure 6:**
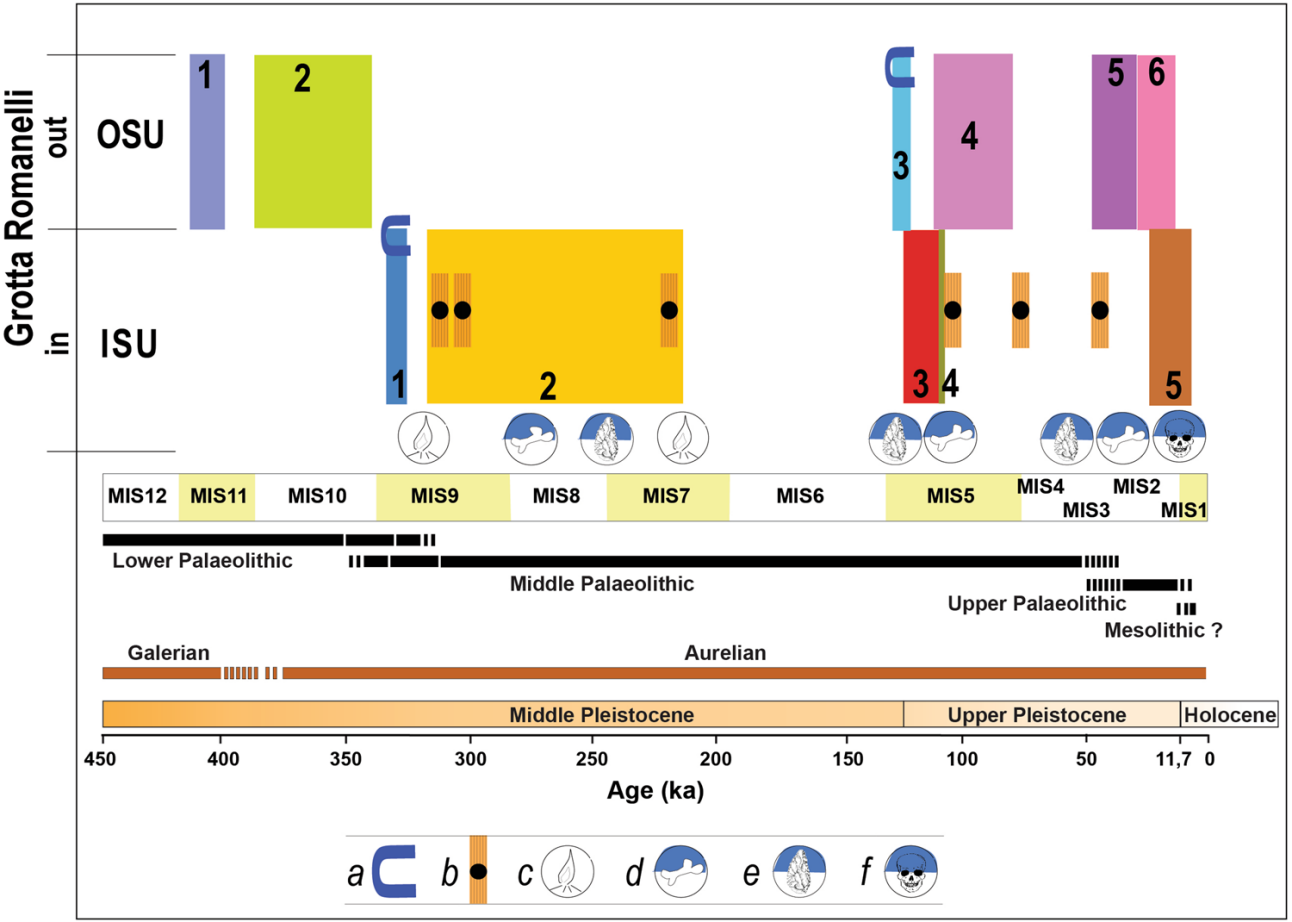
Correlation between MIS chronology and the Stratigraphic Units detected inside (ISU) and outside (OSU) the cave. Legend: a- tidal notch; b- U/Th dated flowstones and stalagmite; c- fireplace in^24^; d- faunal assemblages in^24^ and new excavations; e- lithic tools in^24^ and new excavations; f- human remains and portable art, new excavations^33,58^. Black bars indicate chrono cultural phases, brown bars indicate large mammal biochronology. Artwork by P.Pieruccini and L.Forti.

**Figure 7:**
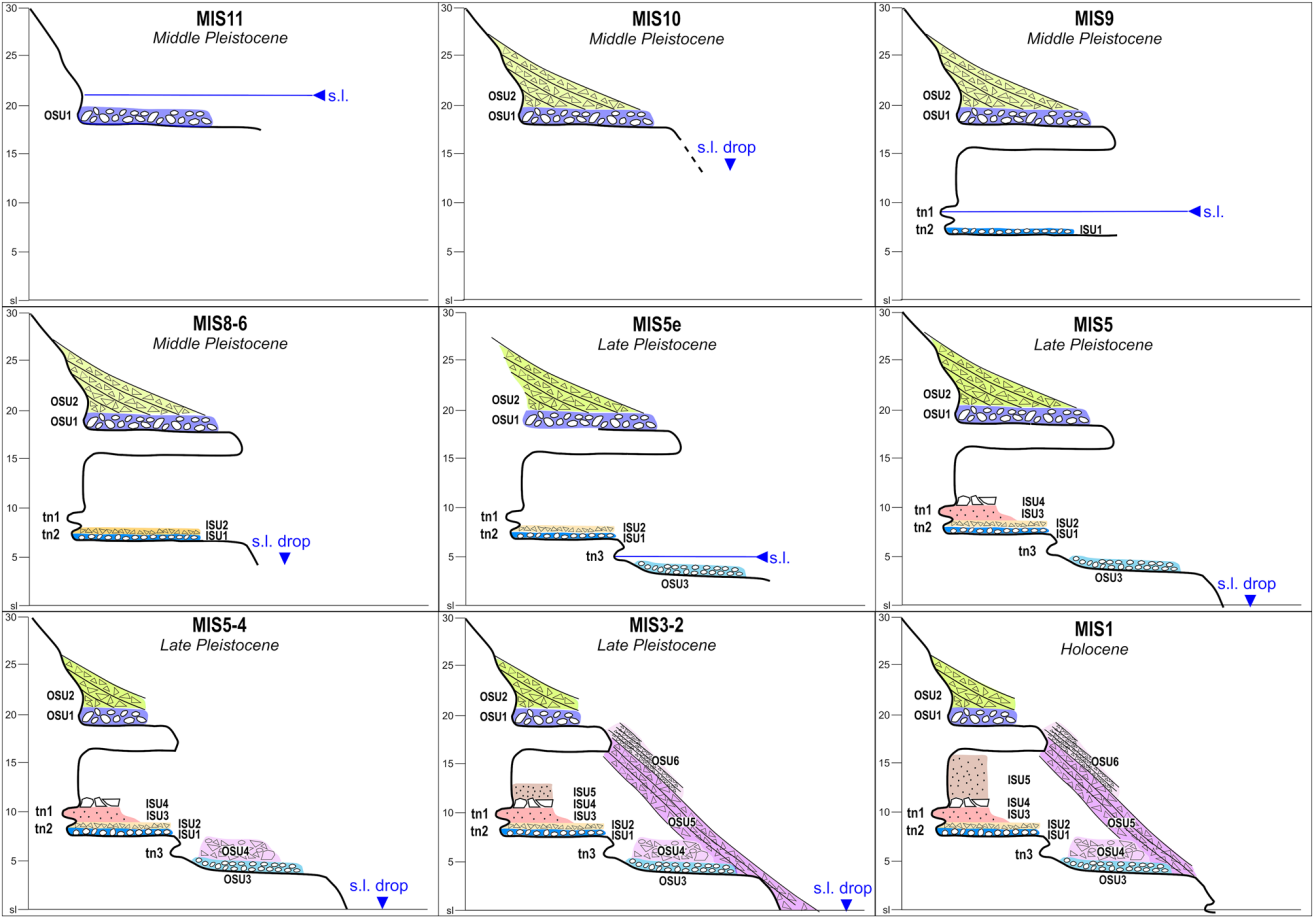
Evolutionary model for Grotta Romanelli and the surrounding cliff. Referred scale in metres a.s.l. Artwork by P.Pieruccini.

Considering this revised litho-, morpho- and chronostratigraphical setting, Grotta Romanelli introduces new elements about the Middle-Late Pleistocene sea-level history of this sector of the Mediterranean. Along the cliff of Grotta Romanelli there are RSL markers vertically stacked at different elevation, indicating 3 marine highstands alternated to as many marine low-stands indicators. The highest RSL marker (Fig. 5a, c), OSU1, indicates a high-energy littoral deposition over a marine abrasion platform modelled during a marine high-stand buried by the OSU2 debris accumulated during a following low-stand. Inside the cave the two tidal notches with *Lithofaga* burrows (tn1 at 9.2 m a.s.l. and tn2 at 7 m a.s.l.) are associated with ISU1. Assuming tn1 and tn2 as belonging to the same transgressive phase^45^ and dated before 320 ka BP (the average lower age of the overlaying low-stand indicator ISU2), they should be associated to the high-stand of the MIS 9 (Figs. 6, 7), as already debated by Mastronuzzi et al.^9^ and Antonioli et al.^11^ based on correlation with the elevation of other marine notches along the tectonically stable Apulian and Italian coasts. This attribution dismantles the Grotta Romanelli paradigm^23^ of the Last Interglacial age for the tidal notches and deposits found within the cave. A further consequence of this chronological setting is the possible attribution of the older marine record represented by OSU1 to the high stand of the MIS 11 (Figs. 6, 7), although currently, a standardised review of pre-MIS 5 Mediterranean sea-level proxies is not available ^54^. The marine terrace linked to the tn3 located at 5.5 m a.s.l. in front of the cave entrance (Figs. 5, 6, 7) can be therefore correlated to the MISs 5e, with an elevation within the variability for the same high stand in the same area of the Mediterranean^9,11,54^. After the Last Interglacial, the sea level drop and climate deterioration were followed by the emplacement of the thick debris units (OSU4, 5 and 6 in Figs. 5, 6, 7) that partially buried the cave entrance and were dismantled during the history of the excavations. OSU4 accumulated on top of OSU3, the MISs 5e terrace, and the chaotic setting as well as the presence of boulders made of cemented stratified breccias, and the occasional presence of rounded pebbles indicates that it originated by rockfalls originated by OSU1 and OSU2 from the overhanging cliff (Fig. 5). The subsequent OSU5 shows a thick layer dipping up to 35 degrees according to the slope, burying both OSU3 and OSU4 and dip below the present-day sea level (Fig. 5), marking its deposition during a well-established lowstand (MIS 4–3). Finally, OSU6, made of finer-grained flake-shaped limestone clasts, suggests the onset of cooler conditions along the slopes and the action of freeze–thaw on bedrock.

Considering the revised chronostratigraphical setting here presented, several implications for large mammal fauna can be inferred. The record of *Stephanorhinus hundsheimensis* (Toula, 1902) in ISU1 and ISU2^35^ is generally documented during the latest Early and Middle Pleistocene sites of Europe (e.g.,^55–57^), with the latest occurrence attested approximately at 0.6 Ma (MIS 16–15;^58,59^). Nevertheless, *Stephanorhinus hundsheimensis* in Grotta Romanelli indicates that this taxon survived in southern Europe until MIS 7 (ISU2, Fig. 6). Moreover, Blanc^23,24^ reported the presence of the straight-tusked elephant, *Palaeoloxodon antiquus* (Falconer & Cautley, 1847), and Hippopotamus *amphibius* Linnaeus, 1758 (Hippopotamus *antiquus* Desmarest, 1882 in Mazza, 1995) in ISU3. This evidence has been deemed as one of their latest occurrences in Europe, allowing to hypothesise their survival up to MIS 4–3^60,61^ although the latest well-dated occurrences of the straight-tusked elephant and hippo are referred to MIS 5^62,63^. The chronological attribution of ISU3 to the early Late Pleistocene supports their European extinction after MIS 5. The complete hemimandible of *Lutra lutra* (Linnaeus, 1758) found during Blanc’s excavations^23,24^ was recently described by Mecozzi et al.^39^ confirming its taxonomic attribution. Since the European record of this carnivoran is quite scarce and mainly known from Holocene deposits^64^, its presence within ISU3 represents one of the earliest evidences of L. *lutra* in Europe.

The most relevant archaeological questions in Grotta Romanelli concerns the sporadic Middle Palaeolithic tools found in ISU1 (2 artefacts) and ISU2 (6 artefacts), whereas a rich sample was collected in ISU3 (1106 artefacts)^31,65^. Almost all the lithics are made on limestone, with the exception of two pieces on quartzite^31,65^. Piperno^65^ described the archaeological sample of the lower complex (ISU1-3), highlighting the use of the Quina technical system, the lack of Levallois technique and the lack of shell tools on *Callista chione*^65^. More recently, Spinapolice^30^ revised the lithic sample from ISU3 and reported the presence of the Levallois technique and in minor part of discoidal method, confirming the use of limestone as a raw material. According to the new age for ISU1 (layer K sensu 20, 21, n = 2) and ISU2 (layer I sensu 20; 21, *n* = 6), the lithic sample from these levels should be attributed to Middle Palaeolithic and Grotta Romanelli becomes one of the oldest evidence of the human technology in Apulia^66^. The abundant lithic from ISU3 represents, instead, one of the oldest Mousterian evidence of Apulia^31,32,66^.

A major issue deriving from the new chrono-stratigraphic setting regards the fireplaces that were described, and completely excavated, by Blanc^23,24^. The lowermost fireplace was found directly resting over the marine gravels of ISU1, inside a large pothole^23^. The fireplace included charcoals, flint tools, and burnt and charred bones of Rhinoceros *merckii* (= *Stephanorhinus hundsheimensis* in^35^), Hippopotamus *amphibius, Dama dama* and *Oryctolagus* cuniculus^23^. A younger fireplace within ISU2 was also reported but lacking any associated lithic tool or bone remains. To date, the use of fire has been recorded in several European sites since 400–300 ka^67,68^, with scarce evidence recorded from caves^68^. In Italy, the oldest evidence is from Campitelli quarry (Tuscany), referred to latest MIS 7^69^ and Molare shelter (Campania), referred to MIS 5^70^. Therefore, according to the new chronology, the fireplace resting above ISU1 represents the earliest record from the Italian Peninsula.

## Final highlights

Grotta Romanelli is one of the few localities in this area of the Mediterranean where 3 RSL indicators consisting of MLPs are stacked along the same cliff at decreasing elevation from 18 m down to 5.5 m a.s.l. (MIS 11, MIS 9, MISs 5e) providing new evidence for Middle-Late Pleistocene sea-level changes in this part of the Apulian region. The newly assessed chronology of the sedimentary succession filling the cave indicates an early frequentation of the cave under conditions of relative environmental stability, dominated by debris accumulation at very low sedimentation rates (ca. 1 m in 150 ka). The oldest human and faunal frequentation occurred between MIS 9 and MIS 7, earlier than previously supposed^21^. The environmental conditions suddenly changed after the Last Interglacial when soil erosion processes on the surrounding slopes brought fine-grained sediments in the cave. A long-lasting hiatus in the sedimentation is marked by the occurrence of several speleothems resting on top of ISU3 and ISU 4 spanning from 112 to 43 ka. The complete and final filling of the cave occurred mostly during the MIS 2–1 transition, recording human and animal frequentation once again. Due to the intense excavation activities of the past and the consequent lack of sediment, we assume that different sedimentary environments and conditions may have characterised the cave sector closer to the entrance, probably providing more suitable conditions for human settling, as indicated by the evidence of fire, food and frequentation previously reported. In fact, in the inner part of the cave the elements of anthropogenic origin are anyhow present (see also, 35) although at a minor extent and as minor components, as evidenced also by micromorphological observations. The new chronostratigraphic assessment of Grotta Romanelli infilling deposit allows to redefine several important bioevents for European mammal palaeo-communities and add important information for human presence in the Mediterranean area. The occurrence of *Stephanorhinus hundsheimensis* (Toula, 1902) in ISU1 and ISU2^35^ represents the last occurrence of this taxon in Europe, previously attested at about 600 ka (Isernia Faunal Unit). This effectively suggests that a large revision of the Middle Pleistocene rhinoceros is needed to clarify the evolution of S. *hundsheimensis*. The attribution of ISU3 to MIS 5 implies that the mandible of the historical collection of Blanc ascribed to the Eurasian River otter, *Lutra lutra* (Linnaeus, 1758)^23,24,39^ is the oldest record of this species in Europe. Moreover, the fossils of *Palaeoloxodon antiquus* (Falconer & Cautley, 1847) and *Hippopotamus amphibius* Linnaeus, 1758 from ISU3 were considered among the last occurrences of these taxa in Europe, allowing to hypothesise their survival up to MIS 4–3^25^. The new chronology of ISU3 suggests that the straight-tusked elephant and the hippopotamus went extinct after MIS 5 ^63^. New insights within the human frequentation of the cave allow the attribution of the fireplaces reported by Blanc^23,24^ as referred to Middle Pleistocene hominins (H. neanderthalensis or Homo *heidelbergensis* Schoetensack, 1908), representing some of oldest fireplaces in Europe Mediterranean area^67,68^. Also, the lithic industry on top of the Middle Pleistocene ISU1 and ISU2 and those from the early Late Pleistocene ISU3 are attributed to the Mousterian^31,65^ and represent the oldest evidence of human technology in the Salentine Peninsula^66^.

## Methods

### Litostratigraphy and facies/microfacies analysis

Lithostratigraphy was investigated by preparing, cleaning, measuring and describing the sections inside the cave and surveying, cleaning, measuring and describing the natural outcrops around and on the cliff just above and below the cave entrance, including those still directly damming the cave entrance. Facies analysis was made at the macroscale in order to get information about the depositional context, the related palaeoenvironment in the surroundings of the cave and the correlation among the different units.

Sedimentary facies in caves are generally based on field description of grain-size, fabric and compositional characteristics of coarse-grained deposits derived by physical and chemical weathering of the bedrock and/or transported by running waters both of karstic and external arrival^66^.

However, when cave sediments are fine-grained or very fine-grained, or with weakly developed sedimentary structures or apparent massive structures, the facies analysis can be improved by their study at a scale smaller finer than the field observation, that is micromorphology of undisturbed samples^71,72^. Moreover, in human settled cave environments the investigation at the micro-scale can be useful for the recognition of evidence such as trampling, maddening, fire-use etc. or depositional and post-depositional processes that can be assessed at the micro scale including palaeo-environmental information of paramount importance for the determination of the sedimentary context of artefacts and other human and animal remains^66,73^.

Undisturbed samples were removed by simple extraction with standard Kubiena boxes (10 × 7 cm) in the field and then prepared at “Servizi per la Geologia” lab (Piombino, Italy) by impregnation with a mixture of resin, styrene, and hardener; curing; cutting into cm‐thick slabs; and final preparation of 25 µm thin sections, measuring 95 × 55 mm (Supplementary Material 2). Thin sections were analysed under a polarising microscope at the University of Torino at magnifications between × 20 and × 1000, using plane polarised and cross polarised light. Images were captured by a digital camera for polarising microscopy. Thin sections were described following the guidelines proposed by Stoops^74^ and after Nicosia and Stoops^75^.

### Geochronology

Six flowstone and one stalagmite samples were dated with U–Th techniques at the High–Precision Mass Spectrometry and Environmental Change Laboratory (HISPEC), Department of Geosciences, National Taiwan University^76,77^. For each sample, clean check subsample, 0.5–1 g, were selected, gently crushed, ultrasonicated, and then dried at 50 °C in a class 10,000 clean room^78^. The cleanest fragments, 0.05–0.10 g, were picked for U–Th chemistry^76^ and instrumental analysis to determine U–Th isotopic and concentration data on a multi-collector inductively coupled plasma mass spectrometer (MC–ICP–MS), Thermo Electron Neptune^77^. Uncertainties in the U–Th isotopic data were calculated offline^79^. Half-lives of U–Th nuclides used for age calculation, relative to 1950 AD, are given in Cheng et al.^80^. Isotopic and age errors given are two standard deviations of the mean and two standard deviations, respectively, unless otherwise noted.

### Morphostratigraphy and geomorphological mapping

Geomorphological and morphostratigraphic investigation were carried on by the classic methods of the field survey in the area surrounding the cave and, on the cliff, above and below the cave entrance. During fieldwork a geomorphological map was sketched by recognising and classifying landforms and related deposits according to their origin (morphogenesis), state of activity (Supplementary Material 3 and Fig. S4 in Supplementary Material) and, at the same time with lithostratigraphic survey, describing the sedimentary characteristics and facies of the deposits. The morpho-stratigraphical position of each Stratigraphic Unit was assessed based on its lithostratigraphic and elevation above the sea level. Similar approach for relative sea-level (RSL) markers such as slope and cave deposits (Terrestrial Limiting Points TLP, following 20) and tidal notches apexes or shoreface deposits (Marine Limiting Points MLP, 20), thus helping to assess different cycles of marine high-stands followed by low-stands. Line drawing sections seaward Grotta Romanelli were realised to underline the chronostratigraphic relationship. The elevation of MLPs (tidal notches apexes and marine terrace below the cave entrance) were measured with total station referred to the ground point control of the archaeological excavations plus its elevation on the present-day tidal notch apex, with an estimated error of ± 20 cm. whereas the elevation of the marine deposits above the cave entrance was measured with GPS and Topo Map, with an estimated ground error of ± 50 cm above the present-day tidal notch apex^81^.

## Supplementary Information

Supplementary Information 1.

## Data Availability

All data generated or analysed during this study are included in this published article (and its supplementary information files).
