# Author Correction: Stratigraphic reassessment of Grotta Romanelli sheds light on Middle-Late Pleistocene palaeoenvironments and human settling in the Mediterranean

**DOI:** 10.1038/s41598-024-69204-x

**Published:** 2024-08-27

**Authors:** Pierluigi Pieruccini, Luca Forti, Beniamino Mecozzi, Alessio Iannucci, Tsai-Luen Yu, Chuan-Chou Shen, Fabio Bona, Giuseppe Lembo, Brunella Muttillo, Raffaele Sardella, Ilaria Mazzini

**Affiliations:** 1 Dipartimento Di Scienze Della Terra, Università Di Torino, Via Valperga Caluso, 35, 10125 Turin, Italy; 2 Dipartimento Di Scienze Della Terra “A. Desio”, Università Degli Studi Di Milano, Via L. Mangiagalli 34. 20133, Milan, Italy; 3 Consiglio Nazionale Delle Ricerche (CNR), Istituto Di Geoscienze E Georisorse, Via G. Moruzzi 1, 56124 Pisa, Italy; 4 Dipartimento Di Scienze Della Terra, “Sapienza” Università Di Roma, Piazzale Aldo Moro 5, 00185 Rome, Italy; 5 Marine Industry and Engineering Research Center, National Academy of Marine Research, Kaohsiung, 806614 Taiwan, ROC; 6 High-Precision Mass Spectrometry and Environment Change Laboratory (HISPEC), Department of Geosciences, National Taiwan University, Taipei, 10617 Taiwan, ROC; 7 Research Center for Future Earth, National Taiwan University, Taipei, 10617 Taiwan, ROC; 8 Museo Civico Dei Fossili Di Besano, Via Prestini 5, 21050 Besano, Varese, Italy; 9 Ministero Dell’Istruzione, Ferrara, Italy; 10 Dipartimento Di Studi Umanistici, Università Di Ferrara, Corso Ercole I d’Este, 32, 44121 Ferrara, Italy; 11 Consiglio Nazionale Delle Ricerche (CNR), Istituto Di Geologia Ambientale E Geoingegneria, Area della Ricerca di Roma 1, 00015 Monterotondo, Rome, Italy

Correction to: *Scientific Reports* 10.1038/s41598-022-16906-9, published online 8 August 2022

The original version of this Article contained an error in Reference 54,

Cerrone, C., Vacchi, M., Fontana, A. & Rovere, A. Last Interglacial sea-level proxies in the Western Mediterranean. Earth Syst. Sci. Data 10.5194/essd-2021-49 (2021).

now reads:

Cerrone, C., Vacchi, M., Fontana, A., and Rovere, A.: Last Interglacial sea-level proxies in the western Mediterranean, Earth Syst. Sci. Data, 13, 4485–4527, 10.5194/essd-13-4485-2021, 2021.

The original Article has been corrected.

