# Supplementary material for: Stratigraphic reassessment of Grotta Romanelli sheds light on Middle-Late Pleistocene palaeoenvironments and human settling in the Mediterranean
Source: Sci Rep. 2022 Aug 8;12:13530. doi: 10.1038/s41598-022-16906-9 (PMC9358667; doi:10.1038/s41598-022-16906-9)
Supplement: Supplementary file 1 — Supplementary Information 1. [file 41598_2022_16906_MOESM1_ESM.docx]

**Supplementary Material**

**Stratigraphical reassessment of Grotta Romanelli sheds light on Middle-Late Pleistocene palaeoenvironments and human settling in the Mediterranean**

Pierluigi Pieruccini^1^, Luca Forti^2,3*^, Beniamino Mecozzi^4*^, Alessio Iannucci^4^, Tsai-Luen Yu^5,6^, Chuan-Chou Shen^6,7^, Fabio Bona^2,8^, Giuseppe Lembo^9^, Brunella Muttillo^10^, Raffaele Sardella^4^ & Ilaria Mazzini^11^

^1^ Dipartimento di Scienze della Terra, Università di Torino, Via Valperga Caluso, 35, 10125 Turin, Italy

^2^ Dipartimento di Scienze della Terra "A. Desio", Università degli Studi di Milano, Via L. Mangiagalli 34. 20133, Milan, Italy

^3^ Consiglio Nazionale delle Ricerche (CNR), Istituto di Geoscienze e Georisorse, Via G. Moruzzi 1 56124 Pisa, Italy

^4^ Dipartimento di Scienze della Terra, “Sapienza” Università di Roma, Piazzale Aldo Moro 5, 00185 Rome, Italy

^5^ Marine Industry and Engineering Research Center, National Academy of Marine Research, Kaohsiung 806, Taiwan, ROC

^6^ High-Precision Mass Spectrometry and Environment Change Laboratory (HISPEC), Department of Geosciences, National Taiwan University, Taipei 10617, Taiwan, ROC

^7^ Research Center for Future Earth, National Taiwan University, Taipei 10617, Taiwan, ROC

^8^ Museo Civico dei Fossili di Besano, Via Prestini 5, Besano, 21050, Italy

^9^ Ministero dell’Istruzione, Ferrara, Italy

^10^ Dipartimento degli Studi Umanistici, Università di Ferrara, Via Paradiso, 12, 44121 Ferrara, Italy

^11^ Consiglio Nazionale delle Ricerche (CNR), Istituto di Geologia Ambientale e Geoingegneria, Area della Ricerca di Roma 1, Monterotondo (Rome), 00015, Italy

*Corresponding author's e-mail: [luca.forti@unimi.it](mailto:luca.forti@unimi.it); [beniamino.mecozzi@uniroma1.it](mailto:beniamino.mecozzi@uniroma1.it)

**Supplementary Material 1. The history of Grotta Romanelli and the origin of its paradigm.**

Grotta Romanelli was discovered by Ulderigo Botti during the 1870s (1), but the first excavation campaigns were carried out by Paolo Emilio Stasi during the earlier 1900s (2). Stasi identified the upper complex, called “Terre Brune”, and the lower complex, called “Terre Rosse”. Both complexes included artifacts and vertebrate fauna assemblages. Based on lithic artifacts found in the “Terre Brune”, Stasi recognized the first evidence of the Upper Palaeolithic in the Italian Peninsula (2). The paleontological findings from “Terre Brune'' played an important role on the evolution of the European Equinae, since Regàlia established a new species, *Equus hydruntinus,* a name strongly related to the Apulian territory*;* the Latin name *hydruntinus* means from “Otranto” (a town in the Salentine Peninsula) (3). In 1914, Gian Alberto Blanc began pioneering fieldwork activities in the cave, applying scientific systematic methods in archeological/paleontological deposits for the first time (4,5). Blanc described a more detailed stratigraphic sequence confirming the presence of two main complexes, from top to bottom: the “Terre Brune'', consisting of five levels (E-A) bearing Upper Paleolithic artifacts and vertebrate fauna, and the lower complex. There, he identified a sequence formed by the “Terre Rosse” (level G), bearing Middle Palaeolithic artifacts and vertebrate fauna, a stalagmitic layer (level H), a bone breccia (level I), and a beach deposit (level K) referred to the Tyrrhenian Stage (Marine Isotopic subStage 5e) (4,5). These two complexes were separated by the level F, a thick stalagmitic layer (4,5). Following Blanc’s interpretation, the deposition of the sedimentary succession started during the early Late Pleistocene, where the upper levels (E-A) were attributed generically to Upper Palaeolithic. Radiocarbon dating performed at the end of 1950 attributed the “Terre Brune '' to the Upper Pleistocene-Early Holocene boundary (6,7,8,9). G.A. Blanc died on December 31, 1966 (10), and, probably, had no time to publish other considerations on the age of the upper complex and its implications for archaeological and paleontological findings. The two speleothems (levels H and F) were radiometrically dated after Blanc’s death: level H yielded an apparent age of < 69,000 years BP, and level F was dated to 40,000 ± 3250 years BP, today corresponding to part of MIS 4 and MIS 3 (11,12,13). These radiometric data confirmed the chronology of the lower part of the succession previously proposed by Blanc. The fieldwork activities continued up to the 1970s coordinated by Cardini with the support of the Italian Institute of Human Palaeontology (IsIPU) (14). The stratigraphic succession described by Blanc (4,5) and the age of the Grotta Romanelli infilling deposits constituted a reference for the archaeological and paleontological studies in the Mediterranean for about 100 years (15,16) as well as the sea-level changes models for the region, although already debated under the morpho-stratigraphic point of view (20,21) (Fig.S1).

**Supplementary Material 2. Micromorphology**

During the excavations in 2018 and 2019 a total number of 17 samples for thin sections were taken (Fig.S2). Other 10 samples have been taken in 2021 and still under preparation. The preliminary work on the already processed samples focussed on the definition of the microfacies to be associated to the sedimentary facies defined at macro-and meso-scale.

The main microfacies characteristics are summarized below:

*Unsorted mixed with anthropogenic constituents* – This microfacies is found both in ISU 3 and 5 and it is made of unsorted, coarse sands to silts, mainly subrounded to angular quartz grains and subordinately coarse sands sized limestone lithorelicts mixed within a massive to crumby clay-silty groundmass. The anthropogenic constituents are made of burnt, partially burn or unburnt bone and charcoal fragments (micro photo a and b (ISU3), g (ISU5); Fig. S3).

*Plane parallel bedded –* This microfacies is made of alternating coarse- to fine-sands laminae with silty and clay laminae, forming couplets with fining-upward trends (micro photo d (ISU3), e (ISU5), Fig. S3). Occasionally the bedding is emphasized by the presence of elongated fragments of bones or charred vegetal remains (micro photo h (ISU5), Fig. S3).

*Cross-bedded –* This microfacies has the same compositional characteristics of the previous one but with less organic constituents such as bones and charred organic materials. Moreover, it is very common in ISU5 (microphoto f (ISU5), Fig. S3) whereas almost absent for ISU3.

*Biological activity* – This microfacies is found only in ISU3 and it is characterised by abundant rounded or channel- and chamber-like biological voids affecting an unsorted mix of sandy to silty sized quartz grains and limestone lithorelicts supported by a massive clay-silty groundmass. Charcoals and bone fragments are alco common (microphoto c (ISU3) Fig. S3).

**Supplementary Material 3. Geomorphological mapping**

The Geomorphological sketch is based and part of the fieldwork carried out in 2017-2019. The Contour Map is derived by the 1:5000 scale Topographic Map of Regione Puglia (CTR, available for free download at https://pugliacon.regione.puglia.it/services/pubblica/paesaggio-urbanistica/cartografia-ctr-dtm-ortofoto-uds-e-carte-idrogeomorfologiche) . Contour equidistance is 5 m.

The field mapping followed the principles of the Legend for Italian Geomorphological Map (available for free download at <https://www.isprambiente.gov.it/en/publications/technical-periodicals/booklets-series-iii-of-sgi/geomorphological-map-of-italy-at-1-50-000-scale-update-and-additions-to-the-guidelines-of-the-geomorphological-map-of-italy-at-1-50-000-scale-paper-i>) where landforms and deposits are classified following their attribution to the morphogenetical process mainly responsible for their modelling. The fieldmaps have been scanned and georeferenced for drawing in QGIS environment classifying landforms and deposits in an on-purpose designed geopackage.

The mapped landforms and deposits are grouped in:

1. Gravity landforms, such as the complex landslide scarp above the entrance of Grotta Romanelli that bounds also the denudated surface due to sliding and flowing of soil, debris and rock fragments during the main rainfall events. Here the bedrock is bared and its whitish colour strongly contrasts with the greyish colour on the surrounding cliffs and scarps where physical and biological weathering indicate a longer stability. To the east of the main entrance, a rockfall/toppling scarp affected the coastal cliff. The entrance of Grotta Romanelli is characterised by the presence of a degradational scarp slowly retreating following mainly physical weathering and rock degradation.

Coarse to very-coarse grained and cemented debris-slope deposits are preserved above the Grotta Romanelli entrance (OSU2), overlaying shoreface gravels (OSU1), and in front of Grotta Romanelli (OSU4-5-6) overlaying the shoreface gravels (OSU3) found at lower elevation than the cave entrance. For description of their sedimentary characteristics, palaeo-environmental significance and chronology see the text. Due to problems of cartographic representation at the scale of the sketch the subdivision of OSU 4, 5 and 6 is not reported in the map. Also, for their characteristics please refer to the text.

1. Structural landforms, namely selective erosion scarps modelled on the Cretaceous limestone and following the seaward dip of the strata.
2. Coastal landforms, represented by coastal cliffs that bound the bay where Grotta Romanelli opens, interrupted in the inner tip of the bay where gravity phenomena caused the retreat of the original cliff. The shoreface deposits OSU1 and OSU3 bury flat abrasion platforms carved on the bedrock and are bounded seaward by scarps that represent the outer margin of the former marine terraces retreating by means of gravity and marine erosion.
3. Karst landforms, namely the entrance of Grotta Romanelli and the entrance of a minor cave found at almost the same elevation along the cliff to the N-NE.

**Supplementary figures**

**Supplementary Fig. S1**

**
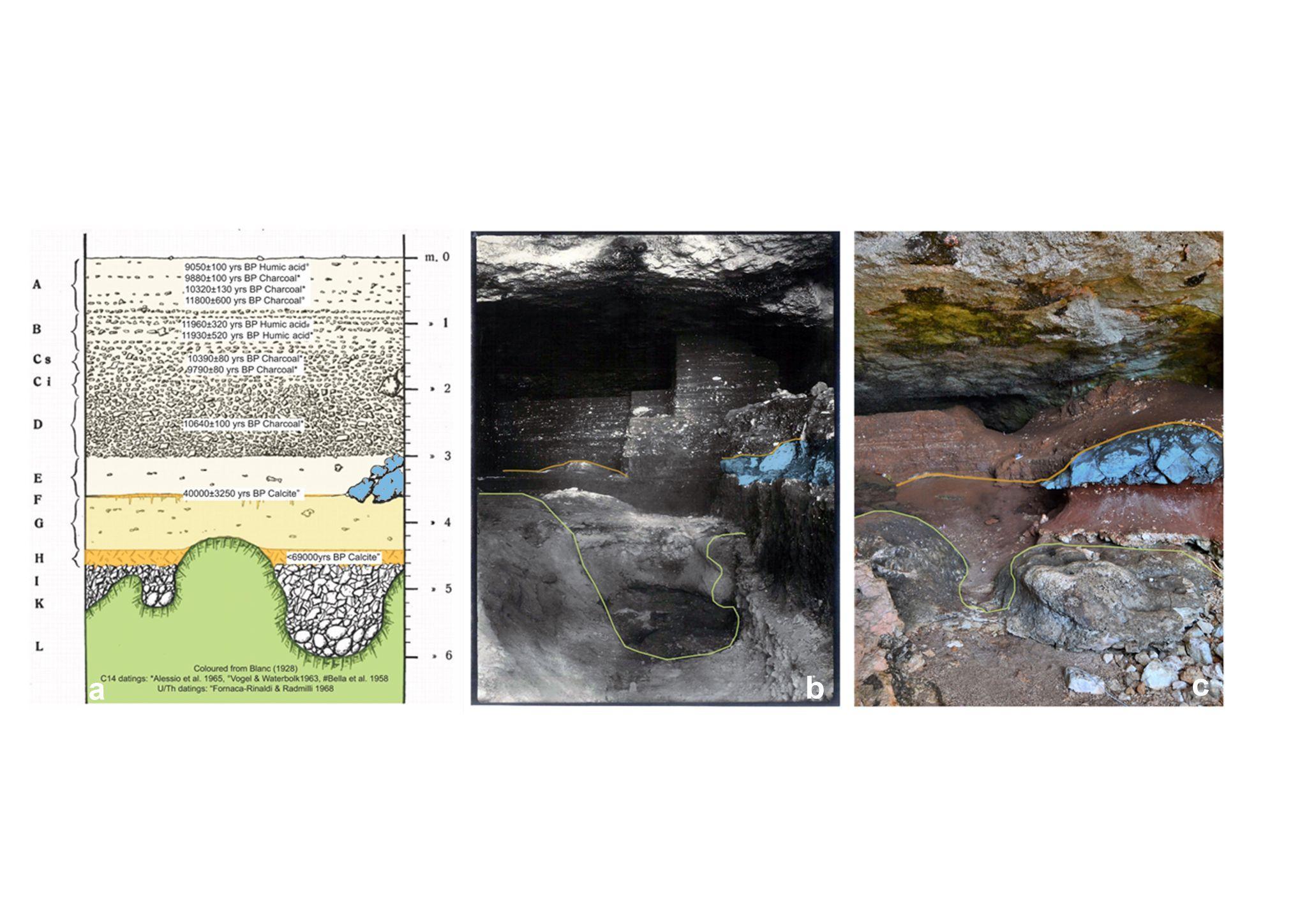
**

**Figure S1.** Stratigraphic log of Grotta Romanelli infilling deposit (modified by 5) with the results of radiocarbon and U/Th dating carried out in previous works (references in the main text) (a). Comparison between the archive photo of 1920 Blanc's excavations (b) and the current situation (c). Modified from (22)

**Supplementary Fig. S2**

**
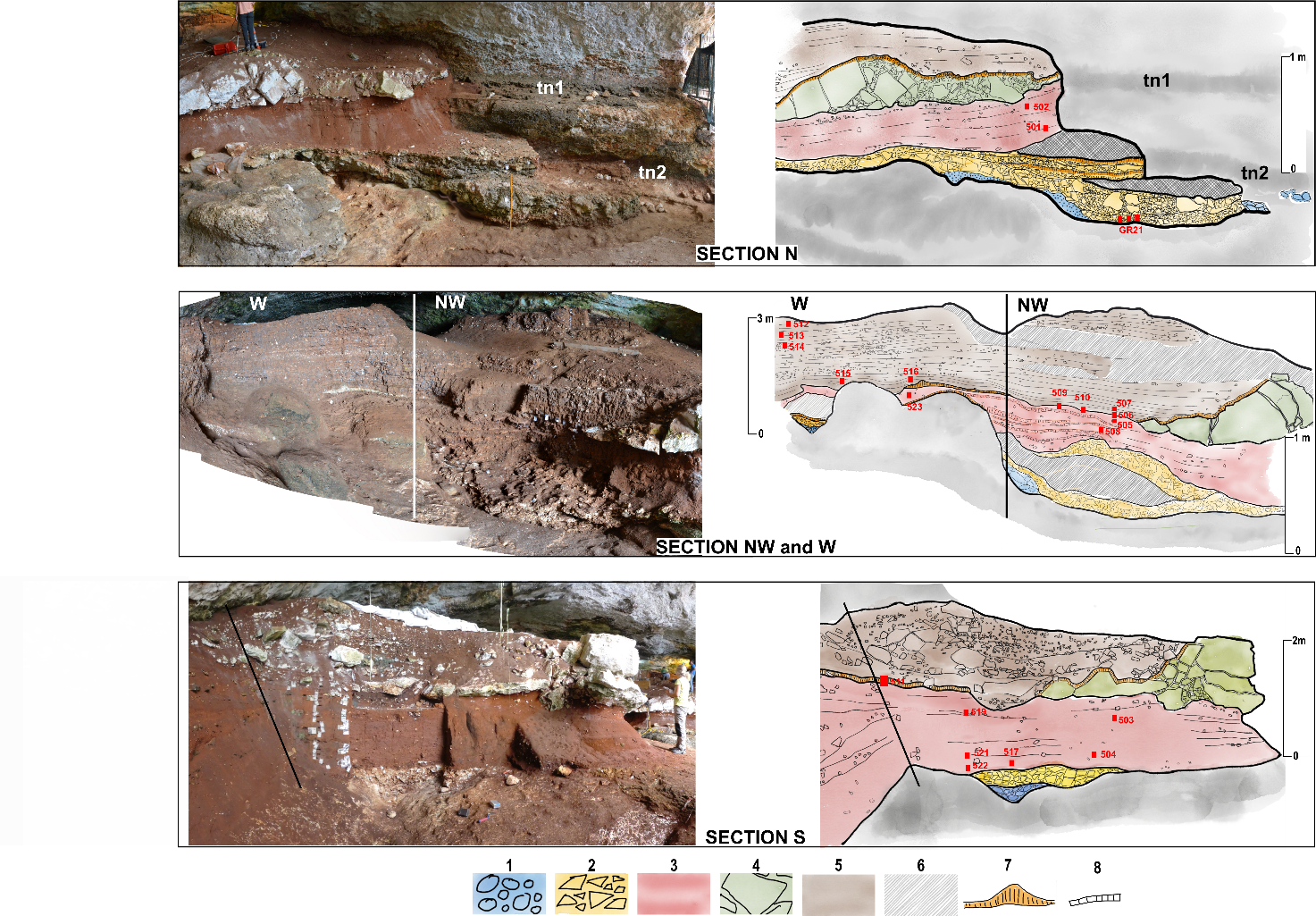
**

**Figure S2.** Positioning of the samples for micromorphology collected during the 2018-2019 excavation campaign. Section North (a); Section North-West and West (b); Section South (c). Legend 1 – ISU1; 2 – ISU2; 3 – ISU3; 4 – ISU4; 5 – ISU5; 6 – covered or plan view; 7 – speleothems; 8 - carbonatic crust (acronyms refer to Table 1 in the main text). Orthophotos by L.Forti and Artwork by P.Pieruccini

**Supplementary Fig. S3**

**
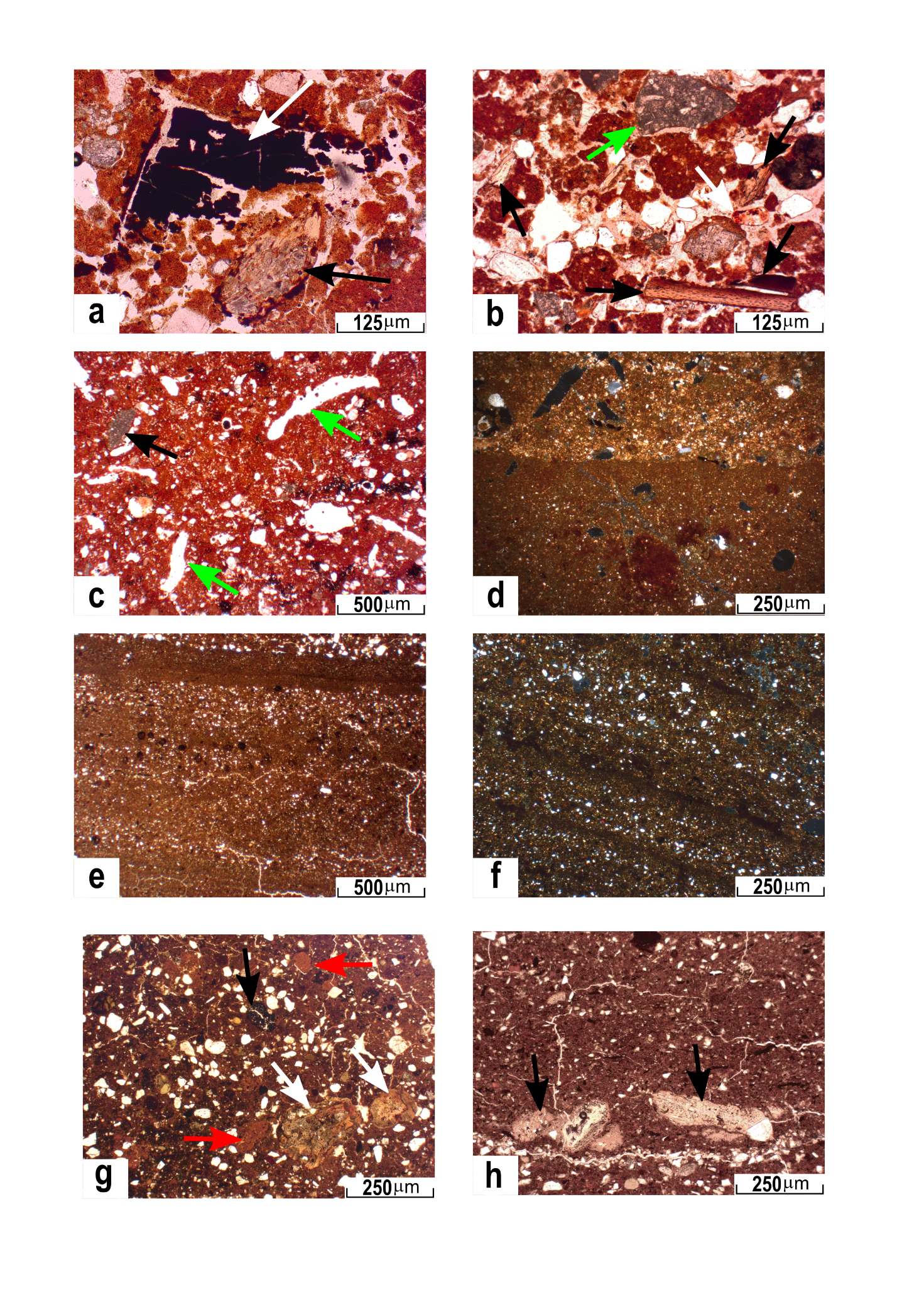
**

**Figure S3**. Selection of microphotographs of thin sections from ISU3 and ISU5. PPL-Plane Polarized light XPL – Cross polarized light. For location and numbering of the samples see Fig. S2.

PPL, ISU3 (504): anthropogenic elements: charcoal fragment (white arrow) and partially phosphatised burnt bone (black arrow) (a).

PPL, ISU3 (501): unsorted mixed microfacies, quartz grains locally burnt (white arrow) with coarser unweathered limestone lithorelicts (green arrow) and burnt bones ((black arrows) (b)

PPL, ISU3 (503): massive microfacies with abundant biological features such as channel and voids (green arrows), rare limestone rock-fragments (black arrow) and blackish Fe-Mn masses (c).

XPL, ISU3 (508): plane parallel bedded laminated microfacies with alternating clays-dominated laminae (bottom) and sandy silty laminae (top) (d).

PPL, ISU5 (506): horizontally bedded laminae (e).

XPL, ISU5 (514): cross-bedded laminated microfacies (f).

PPL ISU5 (516): unsorted massive microfacies with abundant colluviated features derived from the erosion of I-SU3 (red arrows), burnt partially phosphatized bones and phosphatic nodules (white arrows) and rounded charcoal grains (green arrows) (g).

PPL, ISU5 (516): plane parallel bedding locally includes coarse partially burnt bone fragments (black arrows) oriented parallel to the laminae (h). Micromorphological photos by P.Pieruccini

**Supplementary Fig. S4**


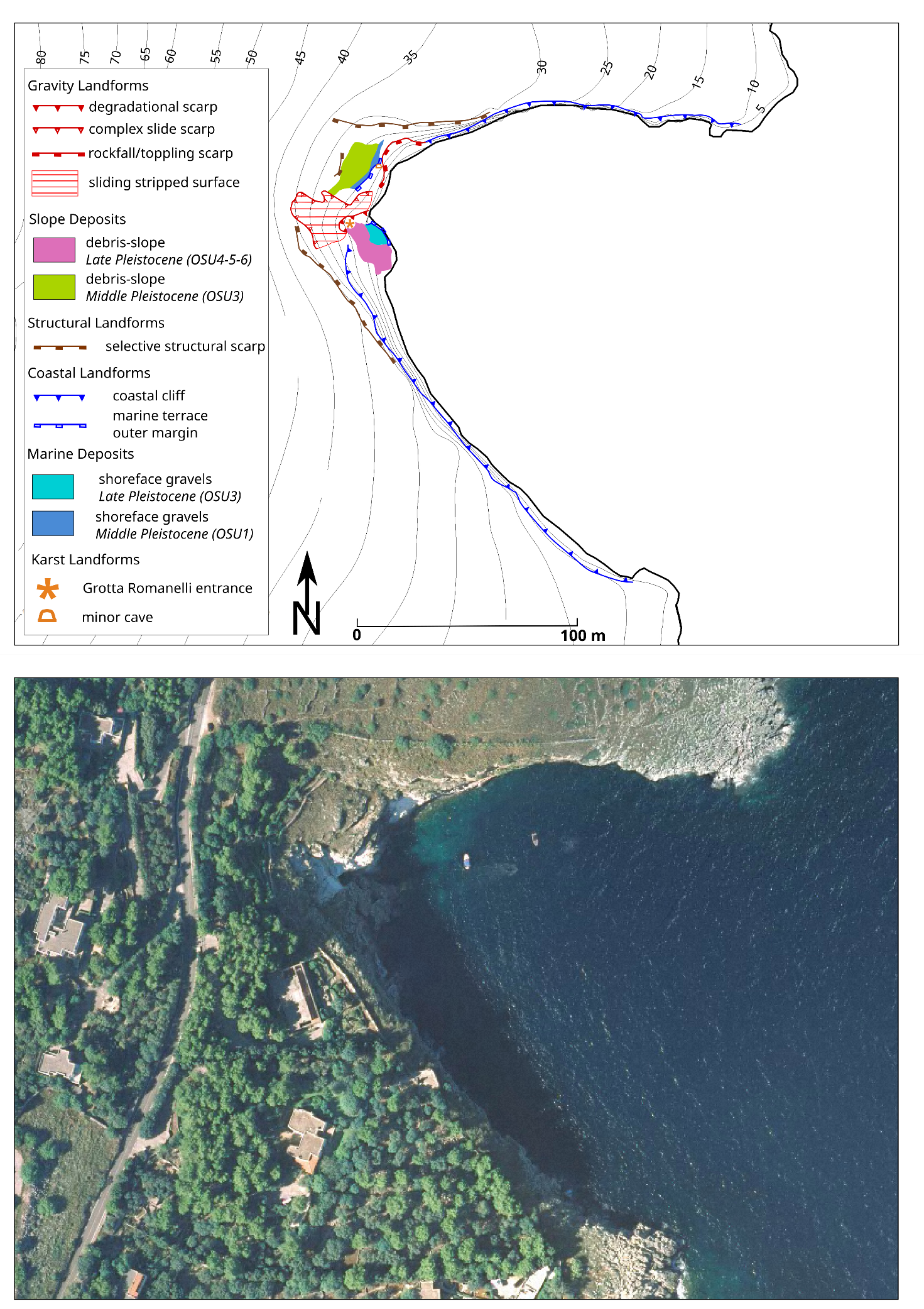


**Figure S4.** Geomorphological sketch of the Grotta Romanelli area (A) and Colour 2006 Aerial Photo (B) of the same area (same scale as the sketch. The orthophoto and the “Carta Tecnica Regionale of Apulia region” were visualized and elaborated as basemap with QGIS 3.16.7 software and free downloaded from <https://pugliacon.regione.puglia.it/services/pubblica/paesaggio-urbanistica/cartografia-ctr-dtm-ortofoto-uds-e-carte-idrogeomorfologiche>). Geomorphological sketch by P.Pieruccini.

**Supplementary Table S1**

| **Sample ID** | **Type** | **Description** | **^238^U** | | **^232^Th** | | **d^234^U** | | **[^230^Th/^238^U]** | | **^230^Th/^232^Th** | | **Age (yr ago)** | | **Age (yr BP)** | | **d^234^U initial** | |
| --- | --- | --- | --- | --- | --- | --- | --- | --- | --- | --- | --- | --- | --- | --- | --- | --- | --- | --- |
|  |  |  | **10-9g/g** *^a^* | | **10-12g/g** | | **measured***^a^* | | **activity***^c^* | | **atomic (x 10-6)** | | **uncorrected** | | **corrected** *^c,d^* | | **corrected** *^b^* | |
| GRN108 | Stalagmite | top ISU4 and bottom ISU5 | 469,4 | ± 1,9 | 0,6748 | ± 0,0062 | -36,1 | ± 5,9 | 0,3157 | ± 0,0048 | 3620 | ± 63 | 43.381 | ± 882 | 43.296 | ± 882 | -40,9 | ± 6,7 |
| GRB345 | Flowstone | top ISU4 and bottom ISU5 | 488,41 | ± 0,58 | 9502 | ± 26 | 4,1 | ± 1,5 | 0,4975 | ± 0,0024 | 421,6 | ± 2,3 | 74.565 | ± 555 | 73.981 | ± 609 | 5,0 | ± 1,8 |
| GRG280 | Flowstone | top ISU4 and bottom ISU5 | 354,60 | ± 0,68 | 6,994 | ± 0,029 | 6,1 | ± 2,1 | 0,6491 | ± 0,0039 | 542,6 | ± 3,8 | 112.790 | ± 1270 | 112.506 | ± 1286 | 8,4 | ± 2,9 |
| GRN172 | Flowstone | top ISU2 | 5572 | ± 11 | 2564 | ± 117 | 52,6 | ± 2,4 | 0,929 | ± 0,044 | 33,3 | ± 2,2 | 223.616 | ± 35468 | 218.820 | ± 34265 | 98 | ± 12 |
| GRG247 | Flowstone | within ISU2 | 924,8 | ± 1,2 | 532 | ± 12 | 49,1 | ± 1,9 | 1,029 | ± 0,028 | 29,5 | ± 1,0 | 365.777 | ± 93882 | 359.965 | ± 87386 | 136 | ± 55 |
| GR17013 | Flowstone | bedrock bottom ISU2 | 1050,5 | ± 1,6 | 301,8 | ± 5,0 | 87,9 | ± 1,8 | 1,062 | ± 0,021 | 60,9 | ± 1,6 | 327.702 | ± 39927 | 325.003 | ± 39053 | 220 | ± 31 |

**Table S1.** Uranium and Thorium isotopic compositions and ages for stalagmite/flowstone samples collected from Grotta Romanelli by MC-ICPMS, Thermo Electron Neptune, at NTU.

Analytical errors are 2s of the mean.

^a^[^238^U] = [^235^U] x 137.818 (±0.65‰) (17); d^234^U = ([^234^U/^238^U] activity - 1) x 1000. ^b^d^234^Uinitial corrected was calculated based on ^230^Th age (T), i.e., d^234^Uinitial = d^234^Umeasured X el234*T, and T is corrected age.

^c^[^230^Th/^238^U] activity = 1 - e-l230T + (d^234^Umeasured/1000)[l230/(l230 - l234)](1 - e-(l230 - l234) T), where T is the age.Decay constants are 9.1705 x 10-6 yr-1 for ^230^Th, 2.8221 x 10-6 yr-1 for ^234^U (18), and 1.55125 x 10-10 yr-1 for ^238^U (19)

^d^Age corrections, relative to 1950 AD, were calculated using an estimated atomic ^230^Th/^232^Th ratio of 1.67 (± 100%) x 10-6.

**Data Availability Statement**

All data generated or analysed during this study are included in this published article (and its supplementary information files).

**References**

1 Botti, U. Sulla scoperta di ossa fossili in Terra d’Otranto. *Boll. Regio Com. Geol.* **1**, 7–8 (1874).

2 Stasi, P. E. & Regàlia, E. Grotta Romanelli stazione con faune interglaciali calde e di steppa. Nota preventiva. *Soc.It. Antropol*. **1**, 17–81 (1904).

3 Regàlia, E. Sull’*Equus* (*Asinus*) *hydruntinus* Regàlia della Grotta di Romanelli (Castro, Lecce) *Arch. Antropol. Etnol.* **37**, 375–390 (1907).

4 Blanc, G. A. Grotta Romanelli I. Stratigrafia dei depositi e natura e origine di essi. *Arch. Antropol. Etnol.* **50**, 1–39 (1920).

5 Blanc, G.A. Grotta Romanelli II. Dati ecologici e paletnologici. *Arch. Antropol. Etnol.* **58**, 1–49 (1928).

6 Bella, F., Blanc, A.C., Blanc, G.A. & Cortesi, C. Una prima datazione con il carbonio 14 della formazione pleistocenica di Grotta Romanelli (Terra d’Otranto). *Quaternaria* **5**, 87–94 (1958).

7.Vogel, J. C. & Waterbolk, H. T. Groningen radiocarbon dates IV. *Radiocarbon* **5**, 63–202 (1963).

8 Alessio, M., Bella, F. & Cortesi, C. University of Rome carbon14 dates II. *Radiocarbon* **6**, 77–90 (1964).

9. Alessio, M., Bella, F., Bachecchi, F. & Cortesi, C. University of Rome Carbon-14 dates III. *Radiocarbon* **7**, 213–222 (1965).

10. Fornaseri M. & Blanc G. A. *La Chimica e l’Industria,* **49**, 3–5 (1967).

11 Fornaca-Rinaldi, G. Il metodo 230Th/238U per la datazione di stalattiti e stalagmite. *Boll. di Geofis. Teor. ed Appl.* **10**, 3–14 (1968a).

12 Fornaca-Rinaldi, G. 230Th/234Th dating of cave concretions. *Earth & Planet. Sci. Lett.* **5**, 120–122 (1968b).

13 Fornaca-Rinaldi, G. & Radmilli, A. M. Datazione con il metodo 230Th/238U di stalagmiti contenute in depositi musteriani. *Atti Soc. Toscana Sci. Nat.* **75** (1), 639–646 (1968).

14 Cardini, L. & Biddittu, I. Attività scientifica dell’Istituto Italiano di Paleontologia Umana della sua fondazione. *Quaternaria* **9**, 385–408 (1967).

15 Sardella, R. *et al.* Grotta Romanelli (Southern Italy, Apulia): legacies and issues in excavating a key site for the Pleistocene of the Mediterranean. *Riv. It. Paleont. Strat.* **124** (2), 247–264 (2018).

16 Sardella, R. *et al*. Grotta Romanelli (Lecce, Southern Italy) between past and future: new studies and perspectives for an archaeo-geosite symbol of the Palaeolithic in Europe. *Geoheritage* **11**(4), 1413–1432.<https://doi.org/10.1007/s12371-019-00376-z> (2019).

17 Hiess, J., Condon, D. J., McLean, N., & Noble, S. R. ^238^U/^235^U systematics in terrestrial uranium-bearing minerals. *Science* **335** (6076), 1610-1614. 10.1126/science.1215507 (2012).

18 Cheng, H. *et al.* Improvements in ^230^Th dating, ^230^Th and ^234^U half-life values, and U–Th isotopic measurements by multi-collector inductively coupled plasma mass spectrometry. *Earth Plan. Sci. Let.* **371**, 82–91. <https://doi.org/10.1016/j.epsl.2013.04.006> (2013)

19 Jaffey, A. H., Flynn, K. F., Glendenin, L. E., Bentley, W. T. & Essling, A. M. Precision measurement of half-lives and specific activities of U 235 and U 238. *Physical Review C* **4**(5), 1889 (1971)

20. Mastronuzzi, G., Quinif, Y., Sansò, P. & Selleri, G. Middle-Late Pleistocene polycyclic evolution of a stable coastal area (southern Apulia, Italy). *Geomorphology* **86**(3–4), 393–408. <https://doi.org/10.1016/j.geomorph.2006.09.014> (2007).

21. Antonioli, F. *et al.* Morphometry and elevation of the last interglacial tidal notches in tectonically stable coasts of the Mediterranean Sea. *Earth-Sci. Rev*. **185**, 600–623. <https://doi.org/10.1016/j.earscirev.2018.06.017> (2018).

22. Forti, L., Mazzini, I., Mecozzi, B., Sigari, D. & Sardella, R. Grotta Romanelli (Castro, Lecce): un sito chiave del Quaternario mediterraneo. *Geologicamente* **2**, 18–27 (2020).
